# Patterns of internet use and mental health of high school students in Istria County, Croatia: cross-sectional study

**DOI:** 10.3325/cmj.2015.56.297

**Published:** 2015-06

**Authors:** Petar Bezinović, Darko Roviš, Nena Rončević, Lovorka Bilajac

**Affiliations:** 1 Institute for Social Research in Zagreb, Zagreb, Croatia; 2 Department of Social Medicine and Epidemiology, Faculty of Medicine, University of Rijeka, Rijeka, Croatia; 3 Teaching Institute of Public Health of Primorsko-Goranska County, Rijeka, Croatia; 4 Department of Education, Faculty of Humanities and Social Sciences, University of Rijeka, Rijeka, Croatia

## Abstract

**Aim:**

To examine associations between different forms of internet use and a number of psychological variables related to mental health in adolescents.

**Methods:**

A cross-sectional survey was carried out on a representative sample of students (N = 1539) from all high schools in the region of Istria in Croatia (14-19 years). The associations between four factors of internet use and nine mental health indicators were analyzed using canonical correlation analysis.

**Results:**

The four canonical functions suggested a significant association between different types of internet use and specific indicators of mental health (*P* < 0.001). Problematic internet use, more typical among boys, was associated with general aggressive behavior and substance abuse (*P* < 0.001). Experiences of harassment, more typical among girls, were associated with health complaints, symptoms of depression, loneliness, and fear of negative evaluation (*P* < 0.001). Using the internet for communication and entertainment was associated with better relationships with peers (*P* < 0.001), while use of the internet for academic purposes was associated with conscientiousness (*P* < 0.001).

**Conclusion:**

The results suggest that different patterns of internet use are significantly associated with specific sets of positive and negative mental health indicators. The data support the assumption that internet use can have both positive and adverse effects on the mental health of youth.

New technologies, and especially the use of computers and the internet, are part of the everyday lives of young people and have a significant impact on their psychological development. Indeed, this mass use of new media technologies presents parents and society with a challenge to protect and support the positive development of children and youth. To date, a number of studies have examined the positive and negative aspects of using internet technologies.

Literature points to several positive aspects of internet use (1,2): for information acquiring, communication, and social networking, entertainment, and online shopping. More specifically, adolescents use the internet as a useful source of information about school assignments, daily events, interests and hobbies, or health and sexuality concerns. In these instances, online activities aimed at connecting with peers have a significant place. Visiting social networking sites and using communication tools such as email, chat, forums, and discussion groups enables the creation of friendships and social groups and contributes to the development of personal identity (3). The use of computers and the internet (cyberspace) has also been argued to provide opportunities for new and faster learning, exercising one's self-control, considering different opinions, expressing one's attitudes and tolerance, and developing skills in critical thinking and decision-making (4). Best et al (5) found that use of online communication technologies contributed to increased self-confidence, better perception of social support, greater social capital, positive experimenting with one’s own identity, and greater opportunities for open self-disclosure. Conversely, adolescents who do not use the internet might trail behind in the development of such positive attitudes and traits and risk being rejected by their peers (6). Finally, Livingstone et al (7) found that a certain amount of risk exposure was useful in building resilience.

In contrast to these positive influences of internet use, harmful effects of internet abuse range from exposure to inappropriate sexual content, pornography, and violence (2,7) to humiliation and cyber-bullying (6,8-10) and internet addiction (11,12). Research has confirmed the link between internet abuse and social isolation, depression and, anxiety (1,5,13,14), alcohol and drug abuse and gambling (15), and problems with physical health (16). Ybarra and Mitchell (17) found a connection between experiences of threats or humiliation in the virtual world and absences from school, lower school achievement, substance use disorders, delinquency and depression. Fekkes et al (18) point to the association between victimizing experiences and a number of physical, emotional, and behavioral problems, such as headaches, tension, fatigue, loss of appetite, enuresis, and sleeping problems. Slonje and Smith (19) define such experiences as a product of cyber-bullying and argue that this can be viewed as another form of aggressive behavior. Gender has emerged as a significant predictor of the manner in which the internet is used. Specifically, girls tend to experience victimizing experiences, while boys more frequently demonstrate antisocial behavior (20,21).

Although many studies have identified both positive and negative correlates of internet use, there have been only a few studies using complex multivariate analyses to identify broader patterns of internet use and adolescent mental health (22,23). Less is known about how adolescents exhibiting different personalities and different emotional and behavioral patterns engage in internet use and what might be the consequences of this engagement. The aim of this study was to determine the specific patterns of internet use and mental health among adolescents. The following hypotheses were tested:

H1: Problematic internet use is associated with externalized symptoms and other negative indicators of mental health. Exposure to victimizing and disturbing content on the internet is associated with internalized symptoms and negative indicators of mental health.

H2: Prosocial internet use (aimed at connecting with peers and entertainment) and internet use for school purposes are associated with positive aspects of mental health.

H3: Problematic internet use is present more often in young men, while exposure to disturbing content on the internet is more frequently experienced by young women.

## Methods

### Study population

The study was conducted in December 2013 on a representative sample of all high schools (22 schools) in Istria County, Croatia. A proportional stratified sample of students was drawn from the first to fourth grade from every school. A sample of 20% of students was randomly chosen from each generation by using the Research Randomizer program (24). The study involved N = 1539 students, 772 (50.1%) male and 767 (49.9%) female. The mean age of participants was 16.26 years (standard deviation = 1.187 years).

### Study design

A cross-sectional survey was carried out as part of a pseudo-longitudinal monitoring study of the quality of life and risk behaviors exhibited in the population of secondary school students in Istria (25-28). The results of this study have been compared with previous studies and have been used in public health policy planning and the development of targeting strategies for prevention of risky behaviors in regional high schools.

### Data collection

The survey was conducted in accordance with the Croatian Code of Ethics of conducting research with children (29). Ethical approval was received from the schools’ founder (Istria County) as a competent administration authority. The study was carried out in schools, in groups of up to 25 students. The participants were informed about the purpose and objectives of the research prior to questionnaire administration. They were also informed that participation was voluntary and anonymous. None of the participants refused to participate. The survey was administered by specially trained students from the University of Pula.

### Measures

The questionnaire used for this study was designed to examine the students' experience of computer and internet use and to assess various aspects of psychological functioning, including some mental health symptoms.

### Internet use

Internet use was measured using 23 items. Participants rated each item using a four-point Likert-type scale (1 = Never, 2 = Rarely, 3 = Often, 4 = Very often). Given that our internet use scale is a new measure, we performed an exploratory factor analysis on the set of items to define the main dimensions of the scale. A four-factor structure was found with factors associated with communication/entertainment, usage of internet for academic purposes, problematic internet use, as well as the online harassment experience (Table 1). Similar categories of internet use and items were used in other studies (2).

**Table 1:** Factor loadings for patterns of internet use items (rotated factor solution)

	Component			
	1	2	3	4
**Use for communication/entertainment**				
Using a computer for communication	0.81			
Spending time on the internet	0.79			
Using a computer to listen to music	0.71			
Using a computer for fun	0.70			
Visiting Facebook, MySpace, Twitter, and the like	0.66			
Using the computer to search for information	0.61	0.31		
Using a computer for file sharing (file sharing, download, upload)	0.58			
Surfing the internet with no particular goal	0.56		0.37	
Using the internet for chat or instant messaging	0.51			
**Use for academic purposes**				
Looking for literature on the internet		0.67		
Performing online tasks that we get for homework		0.64		
Visiting the school website		0.63		
Communicating by e-mail with classmates in connection with teaching assignments		0.62		
Using a computer for school purposes	0.41	0.54		
Communicating by e-mail with teachers		0.54		
Communicating via MSN/ICQ or similar with classmates in connection with teaching assignments		0.48		
**The problematic internet use**				
I lied about my age on the internet			0.73	0.30
I gave false information about myself on the internet			0.73	0.32
I am watching pornographic sites			0.62	
I download unauthorized materials (programs, music, movies, etc.) from the internet	0.38		0.59	
**The Harassment Experience Subscale**				
I experienced Internet bullying				0.79
I was asked for my personal or intimate details by unknown persons				0.76
I often find disturbing content on the internet				0.74

The communication/entertainment subscale consisted of nine statements measuring the frequency of internet use for social networking and recreational purposes. The internal consistency (Cronbach alpha) of this subscale was α = 0.85. The academic purposes subscale consisted of seven statements measuring the frequency of internet use for completing school tasks. The internal consistency of this subscale was α = 0.72. The problematic internet use subscale consisted of four statements measuring the frequency of false representation and online behavior typically not allowed to minors. The internal consistency of this subscale was α = 0.67. The online harassment experience subscale consisted of three statements examining the effect of internet use on the user's sense of mental well-being or safety. The internal consistency of this subscale was α = 0.73.

### Self-rated mental health

Self-rated mental health was assessed using an instrument designed to examine both problematic and positive areas of psychological functioning related to mental health and quality of life. The form consisted of nine short subscales measuring six problematic areas: symptoms of depression, health-related complaints, aggressive behavior, social anxiety, feelings of loneliness, substance use, and three “positive” or preventive areas of psychological functioning: friendship, conscientiousness, and life satisfaction. The participants rated each item on the same 4-point scale as for the internet use (1 = Never, 2 = Rarely, 3 = Often, 4 = Very often).

Symptoms of depression were measured using four items representing the symptoms outlined in the Diagnostic Statistical Manual–IV (30). The items were: “I feel tired and drained;” “I feel sluggish and slow;” “I feel nervous and anxious;” “I feel dejected and sad.” The internal consistency of this subscale was α = 0.77.

Adolescent health complaints were measured using a seven-item symptom checklist developed by Haugland and Wold (31) and adapted by Bezinović and Tkalčić (26). The assessed symptoms were: headache, pain in the neck or shoulders, susceptibility to cold (stuffy nose, sore throat), dizziness, gastric problems (nausea, abdominal cramps, heartburn), sleeping difficulties, indigestion (diarrhea, constipation). The internal consistency of this checklist was α = 0.74.

Manifest aggressive behavior was assessed with a six-item scale measuring the frequency of direct physical and verbal aggression involving hurting or harming others (eg, “I intentionally physically assaulted people;” “I participated in group fights;” “The people I am hanging out with are aggressive;” “I insult people, I tell them they're stupid, etc.;” “I destroy things deliberately;” “I swear and yell in public places.”) The internal consistency of this scale was α = 0.80.

Social anxiety was assessed using four items taken from the Brief Fear of Negative Evaluation Scale (32). The fear of negative evaluation construct consists of feelings of apprehension about others' evaluations, distress over these negative evaluations, and the expectation that others will evaluate one negatively. In this survey, four items were used (“I am afraid others will not approve of me;” “When I talk to someone, I worry about what they may be thinking about me;” “I often worry that I will say or do the wrong things;” “I am afraid of other people noticing my shortcomings”). The internal consistency of this group of items was α = 0.85.

Subjective feelings of loneliness and feelings of social isolation were measured with four items taken from the UCLA Brief Loneliness Scale (33) (“There is no one I can turn to;” “I feel isolated from others;” “I feel as if nobody really understands me;” “I feel I do not belong to the world I live in”). The internal consistency of the scale was α = 0.85.

The frequency of substance use was measured via self-reported frequency of cigarette smoking (tobacco), drinking beer, wine, or spirits, using sedatives (tranquillizers), sniffing (glue, varnish), smoking marijuana or hashish, and using amphetamines (speed, ecstasy). A composite index labeled Substance Use was derived by summing the scores across all substances. The internal consistency of this group of items was α = 0.74.

The Friendship Scale assessed one’s closeness to peers (strength of the attachment or bond that one has with friends). It consisted of eight items (“When I have problems, I can always talk to my friend;” “I feel closeness and loyalty towards my peers;” “Friends give me the necessary support and comfort;” “I talk openly about everything with my peers;” “For me, honesty is crucial for friendship;” “My friends respect me;” “I feel comfortable in the company of my classmates;” “People like to hang out with me”). The internal consistency of this scale was α = 0.84.

The Conscientiousness Scale consisted of five items measuring the tendency to follow socially prescribed norms for impulse control, to be task- and goal-directed, to be planful, delay gratification, and follow norms and rules (“I am a well-organized and practical person;” “I am neat and accurate;” “I prefer to do my chores promptly, rather than to think about them;” “I tend to work hard when I need to do something;” “I am persistent, I do not give up easily”). The internal consistency of the scale was α = 0.76.

Satisfaction with Life was assessed using a four-item scale measuring global cognitive judgments of satisfaction with one's life (“I am happy and satisfy with my life;” “Life brings me a lot of pleasure;” “I generally feel good;” “My life has a purpose and meaning”). The internal consistency of this group of items was α = 0.85.

### Statistical analysis

Statistical analyses were performed by using IBM SPSS Statistics Version 20.0 (Armonk, NY; USA). Test of factorial validity of the internet use subscales was performed by exploratory factor analysis (Principal Components Method with Varimax orthogonal rotation and the Scree plot test for determining the number of factors). The internal consistency of all the scales was evaluated using the Cronbach alpha coefficients. Mean differences of study variables between boys and girls were tested by independent samples *t* test. The Pearson bivariate correlations were calculated to test the associations between internet use factors and mental health variables. Canonical correlation analysis (CCA) was used to test the associations between sets of internet use factors and self-rated mental health variables.

## Results

With regard to internet use, boys were typically found to be more prone to use unauthorized content and falsely identify themselves on the internet (*P* < 0.001), while girls were significantly more likely to use the internet for meeting academic obligations. With regard to psychological variables, boys expressed overt aggression significantly more often than girls (*P* < 0.001), while girls reported significantly more health complaints and symptoms of depression (*P* < 0.001). However, girls were also significantly more likely to develop better and more honest friendships (*P* < 0.001) (Table 2).

**Table 2:** Descriptive data and gender differences in internet use and mental health indicators*

	Girls (N = 767)		Boys (N = 772)			
	mean	standard deviation	mean	standard deviation	t-value df = 1537	*P*
Communication/entertainment	74.78	20.34	77.22	20.97	-2.27	0.023
Academic purposes	30.38	16.80	25.57	16.80	5.56	<0.001
Online harassment experience	28.02	24.00	23.07	22.64	4.15	<0.001
Problematic internet use	18.86	15.97	36.83	22.22	-17.88	<0.001
Friendship	74.93	16.57	65.88	18.81	9.84	<0.001
Life satisfaction	69.24	19.48	71.42	18.91	-2.21	0.027
Conscientiousness	61.48	19.22	59.41	19.24	2.09	0.037
Symptoms of depression	42.22	20.11	29.88	18.02	12.60	<0.001
Fear of negative evaluation	41.92	23.55	33.32	21.42	7.44	<0.001
Health complaints	37.63	15.79	27.01	15.70	13.09	<0.001
Index of substances usage	24.26	15.57	27.22	16.73	-3.54	<0.001
Loneliness	22.01	21.60	17.17	18.94	4.63	<0.001
Aggressiveness	12.90	13.25	20.73	18.24	-9.59	<0.001

*For easier comparison, the results on all variables were linearly transformed to a standard scale ranging from 0 to 100.

Given the observed gender differences, the gender variable was also included in the set of mental health indicators in the CCA, which examined the associations between internet use factors and self-rated mental health variables. CCA identifies unique orthogonal canonical functions, which allowed us to establish whether a set of patterns of internet use was associated with clusters of mental health indicators (Table 3). The full CCA analysis yielded four orthogonal functions with squared canonical correlation coefficients (*Rc^2^*) of 0.380, 0.138, 0.061, and 0.023 for each successive function. These squared canonical correlation coefficients provide an estimate of the amount of shared variance between the respective canonical variates. The full model, represented by all four functions, was statistically significant (χ^2^[40]=850.96; *P* < 0.001; Wilks' λ (lambda) = 0.49) with an effect size of 1 – λ = 0.51, thus explaining 51% of the total shared variance between the canonical variates. Although all four canonical functions were statistically significant, only the first two accounted for a satisfactory percentage of variance, where the first function accounted for 38% of the variance and the second for 13.8%. The third and fourth canonical functions jointly accounted for only 8.4% of the variance (Table 4).

**Table 3:** Correlations between patterns of internet use and gender and mental health variables

	Communication/entertainment	Academic purposes	Online harassment	Problematic Internet Use
Gender	0.08^†^	-0.16^†^	-0.10^†^	**0.43^†^**
Symptoms of depression	0.10^†^	0.09^†^	**0.26^†^**	0.02
Health complaints	0.07^†^	0.08^†^	**0.23**^†^	-0.01
Aggressive behavior	0.13^†^	-0.12^†^	0.22^†^	**0.47^†^**
Fear of negative evaluation	0.02	0.09^†^	0.20^†^	0.02
Loneliness	-0.01	0.00	**0.26^†^**	0.06*
Index of 8 substances used	0.18^†^	-0.07*	0.12^†^	**0.31^†^**
Friendship	0.17^†^	0.18^†^	0.01	-0.07*
Conscientiousness	-0.03	0.18^†^	-0.02	-0.13^†^
Satisfaction with life	0.03	0.07*	-0.11^†^	-0.05

*Correlation is significant at the 0.05 level (2-tailed).

†Correlation is significant at the 0.01 level (2-tailed); correlation coefficients greater than 0.25 are in bold.

**Table 4:** Canonical factor structure for functions 1 to function 4*

	Function 1 r_s_	Function 2 r_s_	Function 3 r_s_	Function 4 r_s_
Factor structure, internet use:				
Communication/entertainment	-0.259	-0.248	**0.896**	-0.263
Academic purposes	0.251	-0.311	**0.566**	**0.721**
Online harassment	-0.176	**-0.972**	-0.120	-0.098
Problematic internet use	**-0.953**	-0.113	0.152	0.237
Factor structure, mental health:				
Gender	**-0.732**	**0.495**	0.053	0.282
Symptoms of depression	0.023	**-0.722**	0.110	-0.280
Health complaints	0.045	**-0.743**	0.101	-0.323
Friendship	0.184	-0.141	**0.864**	0.023
Aggressiveness	**-0.778**	-0.369	-0.133	0.022
Fear of negative evaluation	0.021	**-0.558**	-0.083	0.223
Conscientiousness	0.291	-0.066	0.195	**0.784**
Life satisfaction	0.094	0.229	0.369	0.328
Loneliness	-0.073	**-0.641**	-0.380	-0.086
Index of substances use	**-0.528**	-0.181	0.275	-0.395

*rs – structure coefficients greater than 0.45 in bold.

The first canonical function describes the syndrome of problematic behavior. This form of internet use was associated with aggressive behavior and use of addictive substances, behaviors more characteristic for boys than girls. The first function accounted for a significant percentage of 38.0% of the variance (*P* < 0.001).

The second canonical function, which describes feelings of victimization, harassment, or threats via the internet, was associated with the expressed symptoms of depression, health problems, feelings of loneliness, and social anxiety. These manifestations were more characteristic for girls than boys. The second function accounted for a significant percentage of 13.8% of the variance (*P* < 0.001).

The third canonical function, which describes the shared variance of recreational internet use and internet use for academic purposes, was associated with friendship as a measure of positive social relationships. This function accounted for only 6.1% of the variance.

The fourth canonical function applies exclusively to the use of the internet for academic purposes and was associated with conscientiousness as a personality trait. This function accounted for only 2.3% of the variance and was practically negligible.

## Discussion

The CCA revealed an association between patterns of internet use and specific sets of mental health indicators. The first canonical function refers to the syndrome of problem behavior, in this case characterized by false identification on the internet, downloading unauthorized content, and watching pornographic sites, as well as aggressive behavior and alcohol abuse in real life. This finding confirms the first hypothesis that problematic forms of internet use are associated with negative indicators of mental health, and primarily with externalized symptoms. This is consistent with previous research arguing that problematic internet use can be treated as yet another manifestation of the symptoms of externalized behaviors such as aggression and alcohol/drug abuse, which are themselves associated with negative personality characteristics and which jeopardize the mental health of young people (22). Arguably, problematic online behavior can, therefore, be considered an extension of the syndrome of problem behavior typical of adolescence as described by Jessor and Jessor in their theory of problem behavior (34).

The second canonical function describes the victimization experience, ie, harassment and threats on the internet, which was significantly associated with internalized symptoms of depression, anxiety, loneliness, and health complaints, thus corroborating the second part of the first hypothesis. Other studies have similarly confirmed that the experience of online victimization and cyber-bullying in particular, is associated with a range of emotional and behavioral difficulties normally characteristic of actual peer bullying. For this reason, reported feelings of loneliness, helplessness, depression, and anxiety can be viewed as the psychological effects of victimization (6,17,35). On the other hand, a recent review from Livingstone and Görzig (7) indicates that findings of numerous studies, clinical reports, policy analyses, and analyses of criminal cases support the assumption that the probability of an individual being exposed to victimization on the internet is in direct relation to the risks present in the real world. In this sense, generalized feelings of loneliness, social anxiety, depression, and health problems may well be regarded as a risk for internet victimization. In fact, it appears that those who are vulnerable to victimization in the real world become victims in the virtual one as well (7,9).

Together with broader considerations on the impact of the internet (1,4), these considerations seem to support the argument that individuals who are generally prone to internalize problems are more likely to be exposed to victimizing experiences on the internet and even, perhaps, to develop a dependence on the internet, given that they use it for the purpose of regulating their internal states. Likewise, individuals who present externalizing symptoms will be more likely to use the internet for downloading unauthorized content, false self-identification, and harassment of others. Together, these two canonical functions accounted for 51.8% of the variance and represent significant patterns of associations between internet use and mental health. It is important to note that our results confirmed the syndrome of problematic online behavior to be more characteristic for boys while victimizing patterns were more characteristic for girls. Together, these findings confirm the set hypotheses and the premises of the aforementioned theories.

The third and the fourth canonical functions describe internet use for the purpose of communication/entertainment and for school purposes, respectively, and are thus associated with positive indicators of mental health, positive social relationships, and a higher level of conscientiousness as personality traits. Although they accounted for a lesser proportion of the variance, these two canonical functions describe potentially interesting patterns of association between internet use and mental health. Namely, these findings suggest that communication technologies may have some positive effects because they enable faster and easier communication, networking, friendship-making, and completion of school tasks (1). Such use of the internet satisfies the needs of adolescents in their overall social development (3) and may be particularly useful for those who have difficulties establishing real-life friendships (36). However, Campbell et al (36) argue that, while those who frequently use chatting claim that they actually benefit from this activity, young people who spend more time on the internet are lonelier and have a higher risk of developing an addiction. While internet use for the purpose of communication is associated with lower levels of depression among adolescents with poor-quality friendships, surfing the internet in the absence of any communication with others can contribute to symptoms of depression and social anxiety.

Using the internet for academic purposes is a productive mode of internet use and is also associated with positive indicators of mental health. Tsitsika et al (23) describe the protective effect of the the school as a place from which the internet is accessed, a finding consistent with numerous studies that emphasize the central role of the school in the personal and social development of adolescents (37,38). In this regard, it is important to stress that the exceptional role played by the school in the lives of children and youth expands to the sphere of digital educational resources and virtual interactions, thus confirming the importance of the school and education as a powerful mechanism for behavior regulation and prevention of risky behavior among young people.

Finally, the life-satisfaction variable observed in this study was not linked to any of the typical internet usage patterns. This is consistent with previous research, where the association between internet use and mental well-being has rarely been found (5). It seems that the positive and negative effects of the internet exist only in specific interactions and pathways not reflected in overall life satisfaction. As such, the seriousness and permanence of these effects remain to be investigated (3).

The correlational design of this study points only to a possible association between the observed phenomena. In order to investigate the effects of the internet on mental health in more detail, a longitudinal design should be used. Moreover, given that we observed respondents who, in the virtual world, grouped themselves in specific virtual groups or on specific sites, the grouping effect of such social groups should also be investigated. Another limitation of the study is the fact that some relevant variables such as time spent on the internet or parental control were not measured. Finally, while the first and the second canonical functions accounted for a significant proportion of variance, the third and fourth functions provided only a small hint at other potential associations. As such, future studies should be devoted to the examination of more specific patterns. Future research with wider set of indicators needs to clarify the causality of these findings and should also aim to develop concepts for more meaningful use of the internet.

In conclusion, the results of this study support the argumentation on how internet use can have both positive and adverse effects on the mental health of youth. Our study suggests that different patterns of internet use, pro-social and anti-social, are related to a specific set of indicators of positive and negative mental health among adolescents.
